# Peer Review of “Eye Care Service Use and Associated Health-Seeking Behaviors Among Malawian Adults: Secondary Analysis of the Malawi Fifth Integrated Household Survey 2019-2020”

**DOI:** 10.2196/58361

**Published:** 2024-04-09

**Authors:** Anonymous

**Keywords:** access to health, health service utilization, eye care use, health-seeking behavior, sociodemographic determinant, visual impairment, social support, women empowerment, education, eye care, pediatric, eye, ophthalmology, visual, ECU, eye service, utilization, Malawi, empowerment, health service use, use

*This is the peer-review report for “Eye Care Service Use and Associated Health-Seeking Behaviors Among Malawian Adults: Secondary Analysis of the Malawi Fifth Integrated Household Survey 2019-2020.”*

## Round 1 Review

Potentially an interesting paper [1], but more details are needed in the methods to enable the reader to understand the results.

The English is not good throughout the document, and the writing could be much more succinct and precise.
